# Ameliorating cognitive control in patients with binge eating disorder by electrical brain stimulation: study protocol of the randomized controlled ACCElect pilot trial

**DOI:** 10.1186/s40337-022-00544-7

**Published:** 2022-02-19

**Authors:** Katrin E. Giel, Kathrin Schag, Peter Martus, Sebastian M. Max, Christian Plewnia

**Affiliations:** 1 Department of Psychosomatic Medicine and Psychotherapy, Medical University Hospital Tübingen, Eberhard Karls University Tübingen, Osianderstr. 5, 72076 Tübingen, Germany; 2 Center of Excellence for Eating Disorders, Tübingen, Germany; 3 Institute for Clinical Epidemiology and Applied Biostatistics, Medical Faculty, Eberhard Karls University Tübingen, Tübingen, Germany; 4 University Hospital of Psychiatry and Psychotherapy, Tübingen, Germany

**Keywords:** Binge eating disorder, Cognitive control, Eating disorder, Non-invasive brain stimulation, RCT, tDCS, Treatment

## Abstract

**Background:**

The current first-line treatment for binge eating disorder (BED), which is psychotherapy, is moderately effective in terms of abstinence from binge-eating. Neurobiological evidence suggests that people affected by BED show difficulties along the spectrum of impulsivity, including inhibitory control impairments and highlights the potential of novel treatment approaches directly targeting inhibitory control, including cognitive training approaches and non-invasive brain stimulation.

**Methods:**

ACCElect is a prospective, randomized controlled pilot trial investigating a novel, food-related inhibitory control training combined with transcranial direct current stimulation (tDCS). 40 patients with BED will be randomly assigned to receive the training either combined with verum or with sham stimulation (control condition). The inhibitory control training is based on principles of the antisaccade paradigm and comprises six training sessions over two weeks. Core aims are the investigation of feasibility and clinically relevant effects of a tDCS-enhanced inhibitory control training in BED patients and the establishment of a data basis for a larger efficacy trial. The primary clinical endpoint is binge-eating (BE) frequency in terms of changes in BE episodes four weeks after treatment termination as compared to baseline. Key secondary outcomes comprise ED pathology and general psychopathology, inhibitory control capacities, quality of life as well as acceptability and satisfaction with the intervention.

**Discussion:**

The results of the present trial will contribute to the development of novel neurobiologically informed treatment approaches for patients suffering from BED.

*Trial registration* The ACCElect trial was prospectively registered on October 1, 2020, under the registration number NCT04572087 at ClinicalTrials.gov **(**https://clinicaltrials.gov/ct2/show/NCT04572087).

## Background

Binge Eating Disorder (BED) is the most common eating disorder (ED) in the general population [1, 2]. Psychotherapy is recommended as the first-line treatment for people with BED [3, 4], while cognitive-behavior therapy (CBT) is currently supported by the largest evidence base [3]. Many patients with BED profit from psychotherapy in terms of central treatment goals such as reduction of or abstinence from binge-eating as core psychopathology of BED, improvements in ED pathology and in quality of life. According to a recent meta-analysis, around half of patients treated with psychotherapy achieve abstinence from binge-eating which is maintained over a 12-months period [5]. However, this also means that the other half of patients still suffer from binge-eating episodes when terminating treatment, hence they do not sufficiently benefit from the first-line treatment.

Given the considerable burden associated with BED [6], these numbers highlight the need for improved therapy outcomes which might be achieved by novel treatment approaches, either as stand-alone therapies or in combination with current state-of-the-art treatments. These novel approaches should be informed by translational research into the underlying mechanisms of binge-eating. Neurobiological evidence [7] suggests that people affected by BED show difficulties along the spectrum of impulsivity [8, 9], including altered reward processing [10], impaired inhibitory control [11] and emotion regulation capacities [12], which also involves changes in underlying neurocircuit functioning [7]. For instance, the dorsolateral prefrontal cortex (dlPFC) is involved in response inhibition [13], and patients with BED have demonstrated reduced activation in the dlPFC together with impaired response inhibition performance on a behavioural level [14]. This identifies the dlPFC as a potential treatment target in patients with BED, and one approach to influence brain areas such as the dlPFC entails non-invasive brain stimulation (NIBS).

NIBS entails different techniques which allow for the transcranial influence on the excitability of the brain. Therefore, NIBS can be used to influence brain areas involved, for instance, in inhibitory control processes [15]. One common NIBS approach is transcranial direct current stimulation (tDCS), a technique which applies a weak constant current of one or two mA via electrodes to a specific brain area through the scalp [16]. The current alters the membrane potential of the neurons thus increasing or decreasing the probability of neuronal firing. In simplified terms, anodal stimulation increases and cathode tDCS decreases cortical excitability [15–17]. A recent meta-analysis supports that tDCS exerts significant effects on inhibitory control as assessed by the Stop Signal Task (SST) in healthy and in clinical populations [18].

### Existing knowledge

Recently, NIBS has been used to influence eating behaviour and has been probed in the treatment of eating and weight disorders, while most studies have used tDCS as a technique [15, 17, 19]. Evidence in healthy and sub-clinical groups as well as in people affected by obesity suggests that tDCS and other forms of NIBS can reduce (short-term) food cravings [15], however, studies investigating the potential of NIBS in clinical populations are still very scarce and have mostly focused on patients with anorexia nervosa (AN) [15]. There is to the best of our knowledge only one published study which has probed the use of tDCS in a sample of people affected by BED or sub-clinical BED [20]. In this study, 30 individuals with BED received either a verum or sham stimulation of the right dlPFC with 2 mA [20], and the verum stimulation resulted in reduced short-term food craving and urge to binge-eat in male participants only and in overall reduced caloric intake directly after the stimulation. Of note, this study exclusively used tDCS as an intervention and did not pre-define a primary outcome or planned subgroup analyses.

However, it has to be considered, that tDCS itself does not induce neuronal activity like e.g. transcranial magnetic stimulation and its effects therefore critically depend on concurrent brain activity [21, 22]. This property of tDCS thus allows for targeting specific networks and associated cognitive or behavioral functions [23–25]. Therefore, a targeted combination with training has been put forward as a promising approach to support adaptive plasticity underlying the therapeutic effects of tDCS [16, 26, 27]. Therefore, combinations of tDCS and cognitive training approaches are discussed as a promising new treatment approach [16]. Particularly, tDCS administration to the prefrontal cortex during task training has shown some promising effects in patients with depressive disorder [28–30] and schizophrenia [16, 31]. BED is also associated with dysfunctions in prefrontal brain areas [14] and patients with BED have demonstrated impairments in a range of cognitive tasks, predominantly in the domain of inhibitory control [9]. These tasks can also be used in terms of training tools to enhance cognitive control [19].

In line with this recent development in the field of NIBS, there is one ongoing feasibility RCT probing a food-related cognitive bias modification training combined with tDCS in people with BED [32]. This study targets the right dlPFC with anodal stimulation and the left dlPFC with cathodal stimulation with 2 mA [32]. In the cognitive domain, especially if inhibitory control is demanded, the right dlPFC seems to be crucially involved and performance might be improved by anodal tDCS [33]. Furthermore, it could be shown, that food craving could be reduced by anodal tDCS of the right dlPFC [34, 35]. Besides, there are three further related registered trials, two are using a different NIBS approach in patients with BED which is repetitive transcranial magnetic stimulation (rTMS) [36] (NCT04129970), and a further trial is combining tDCS with nutritional counselling (NCT04226794).

Prior to the present randomized controlled pilot trial (RCT), we have conducted two proof-of-concept (POC) studies to assess underlying mechanisms and feasibility of this treatment approach [37, 38]. In the earlier POC study, we piloted the feasibility and acceptability of a food-specific inhibitory control training which is based on principles of the antisaccade paradigm [39] in patients with BED [38]. The training approach proved to be feasible and acceptable, and participants showed an improved performance regarding inhibitory control towards pictures of high-caloric foods over three training sessions [38]. Within the second double-blind, randomised, sham-controlled POC study, we combined this inhibitory control training with tDCS of the right dlPFC in 31 patients with BED, contrasting effects of 1 versus 2 mA stimulation intensity [37]. We found that this approach was again feasible and acceptable [37]. While there was no overall effect of stimulation on performance measures, decreased latencies for successful response inhibition were found under the 2 mA condition [37], rendering this as the more promising dosage for a treatment intervention, also in line with the ongoing trial on tDCS in BED [32].

### Study aims

Based on (a) emerging neurobiological findings on the role of inhibitory control in the pathology of BED, (b) promising results of previous studies targeting inhibitory control and eating-related outcomes using tDCS and cognitive trainings and (c) data from our POC studies [37, 38], we have designed the present pilot clinical RCT.

The primary aim of the ACCElect trial is to pilot the treatment protocol of six sessions of a food-specific inhibitory control training combined with 2 mA of tDCS to the right dlPFC. This intervention will be compared to training in combination with sham stimulation as a control condition. Specific aims comprise: (a) confirmation of the feasibility of a tDCS-enhanced antisaccade training in BED patients; (b) establishing evidence regarding clinical relevant effects of a tDCS-enhanced antisaccade training to increase cognitive control over eating in BED patients and (c) establishing evidence for a sample size calculation for a larger multicenter RCT on the efficacy of tDCS in increasing cognitive control in BED.

The primary clinical endpoint is binge-eating (BE) frequency as core psychopathology of BED [40] in terms of changes in BE episodes four weeks after treatment termination as compared to baseline. Key secondary outcomes of the trial comprise the investigation of changes in overall ED pathology and general psychopathology, changes in inhibitory control capacities and broadly defined impulsivity, changes in quality of life as well as acceptability and satisfaction with the intervention.

## Methods/design

The present study protocol is reported according to the SPIRIT checklist [41].

### Study design and setting

ACCElect is a single centre clinical pilot double-blind RCT with two parallel arms.

**Fig. 1:**
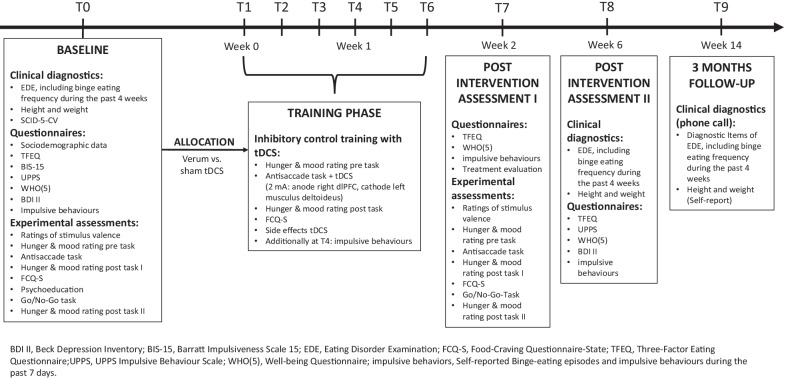
Study procedures and assessment points of the ACCElect trial

Patients are recruited from the outpatient eating disorder service of the Medical University Hospital Tübingen, via study announcements in the media, via email lists of the University Tübingen, and individuals who have previously participated in ED research study at the Department of Psychosomatic Medicine and Psychotherapy and gave written consent to be informed about future studies are also contacted. People who are interested in the study receive verbal and written information material and are screened using a standardized checklist covering inclusion and exclusion criteria. After providing written informed consent, the baseline assessment takes place. After completion of the baseline assessment, patients will be randomized at a 1:1 ratio to one of the two treatment conditions (see below). As outlined in Fig. 1 and Table 1, the outcomes will be measured at baseline (T0), at each training session (T1–T6), at end of treatment (T7) and at two follow-up timepoints, a four-week follow-up (T8) and a 3-months follow-up (T9).

**Table 1:**
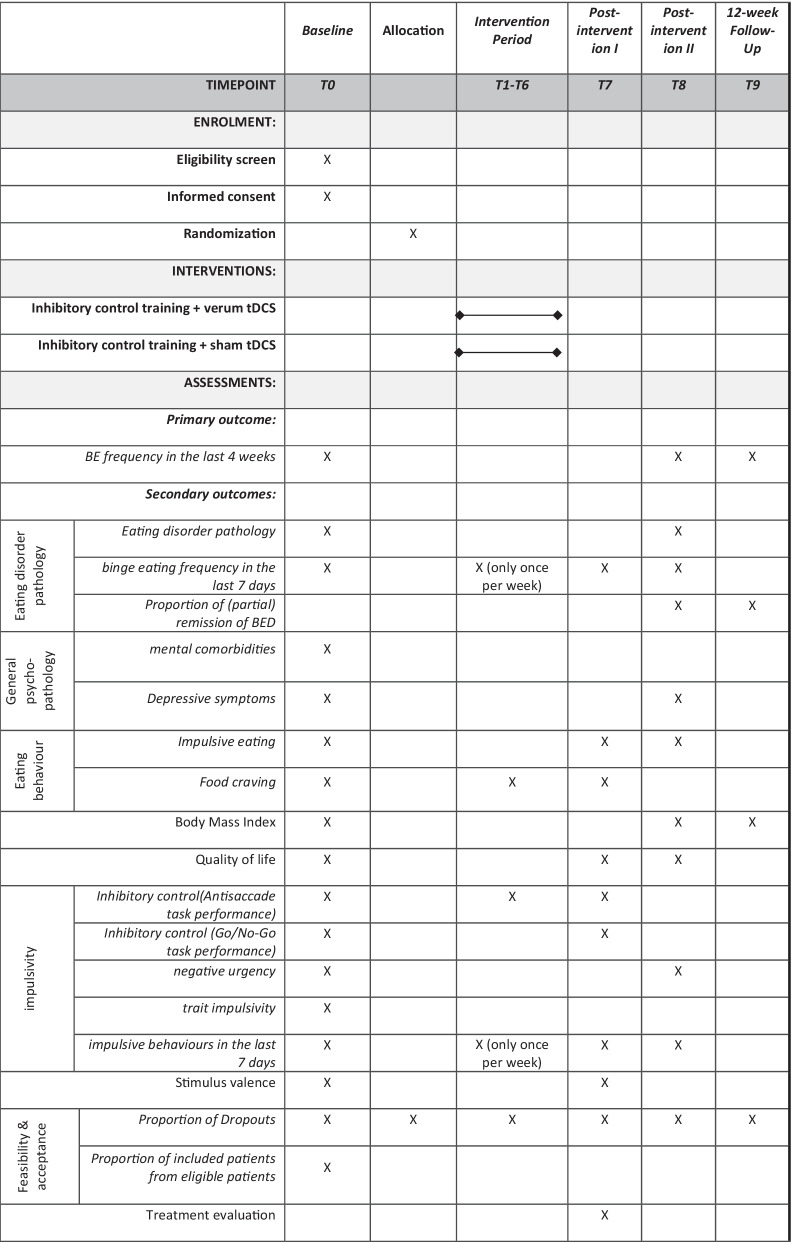
Schedule of enrolment, interventions, and assessments within the ACCElect trial according to SPIRIT

### Study participants and eligibility criteria

The study population consists of individuals who have a diagnosis of a full-syndrome BED according to DSM-5 [42].

#### Inclusion criteria

Patients eligible for the trial must comply with all of the following at randomization:

- Age ≥ 18 years
- BMI > 20 kg/m^2^
- Written informed consent

#### Exclusion criteria

- Insufficient knowledge of German language
- Current pregnancy or lactation period
- Current or lifetime psychotic disorder, bipolar-I disorder, current substance dependence, current suicidality
- Previous bariatric surgery
- Severe somatic diseases which influence weight or eating behaviour (e.g. severe diabetes) and are not controlled by stable medication
- Severe neurologic disease
- Non-removable metal parts in the area of the head (besides metal parts in teeth)
- Pacemaker
- Current intake of neuroleptics and benzodiazepines
- Uncorrected impaired vision, ametropia, eye diseases which prevent from executing the task

### Interventions

Eligible patients receive six sessions of a food-related inhibitory control training, and they will be randomly assigned to receive this either in combination with a verum tDCS of the right dlPFC or in combination with a sham stimulation.

#### Psychoeducation

A short psychoeducation (approximately 5–10 min) is provided by a trained psychologist to all participants, covering an overview on BED, as well as mechanisms of the inhibitory control training combined with tDCS stimulation, and the transfer of the training effects to everyday life.

#### Inhibitory control training

The inhibitory control training is based on the principles of the antisaccade paradigm, an established paradigm requiring prefronal brain activation to suppress prepotent oculomotor reactions to a stimulus presented in peripheral vision [43]. Each trial of the antisaccade paradigm starts with a central fixation cross which is succeeded by a stimulus presented slightly left or right from the cross in a peripheral screen position. The participant is asked to look as quickly as possible upon stimulus onset on the other side of the screen, that is, to perform an antisaccade. We have modified the classical paradigm by using food versus control pictures as peripheral stimuli [38, 39, 44], and found that participants with BED generally had more problems to exhibit antisaccades than BMI-matched obese participants without BED and normal-weight controls, and they had more problems looking away from a food picture they had wrongly looked at [39, 44]. Based on this evidence, the basic idea of the food-specific inhibitory control training is to influence eating behaviour via the improvement of antisaccade performance as a measure of impulsivity. During training sessions, BED patients practice the suppression of a prepotent oculomotor response towards a peripheral food stimulus. On a task level, the aim of the training is the reduction of error rates, that is, the reduction of prosaccades towards food stimuli.

As piloted in the POC study [37], study participants are presented with 40 colour pictures depicting high-caloric food items. The participants rate these pictures concerning valence, i.e. how appetizing the depicted food looks, how much they like the food in general and how much they want to eat it now. Afterwards, the 20 individually highest-rated stimuli are chosen for the training. Each picture is presented four times during one block, while presentation location on the screen (left / right) is counterbalanced. We present the stimuli in four blocks, resulting in 320 trials. Each trial starts with a central fixation cross (1250 ms), succeeded by a 200 ms interstimulus interval. Afterwards, the food picture is presented. After each training session, participants receive feedback about the percentage of errors they have committed.

During task performance, the participant’s gaze behaviour is recorded using a IViewX Hi-Speed eye tracking system (SensoMotoric Instruments, 2010) with a sampling rate of 500 Hz and 0.25°–0.5° gaze position accuracy.

Study participants receive six sessions of the training within 14 days, which is three sessions / week. Hunger and mood are assessed at every training session using visual analogue scales.

#### tDCS

Two 5 × 7 cm electrodes connected to a battery-driven, constant-current stimulator (DC-STIMULATOR MC, NeuroConn GmbH, Ilmenau, Germany) will deliver 15 min of transcranial direct current stimulation (tDCS). The electrodes will be prepared with a Ten20 conductive paste (Weaver and Company, Aurora, CO, USA). An unipolar tDCS montage will be used, placing the cathode extracephalic on the left deltoid muscle and the anode over F4 according to the international 10–20 system of electrode placement [45]. By using an unipolar tDCS montage, solely the right dlPFC should be targeted [46]. After a fade-in of 5 s, the verum group will receive 15 min of 2 mA tDCS, whereas the sham group will only receive 43 s of tDCS before the start of the antisaccade task. This is considered as a valid placebo-condition as typical perceived physical sensations (e.g. tingling) usually fade out in the first 30 s of tDCS [47]. By using a 5-digit number, generated by a person not linked to the study, the stimulator will either apply verum or sham stimulation. Thus both experimenter and participant will be blind regarding the allocated condition.

### Patient safety

After each training, side effects of tDCS will be rated on a scale ranging from 1 (“not at all”) to 5 (“very”): Tingling at the electrode, tingling at other parts of the head, fatigue, slight itching, headache, nausea, other. Other potential (severe) adverse events will be documented by the experimenter.

## Concomitant care

As the present trial focusses on feasibility rather than efficacy, participants are allowed to receive other parallel treatments for their ED. Concurrent use of psychoactive medications is allowed in the trial, with type and dosage of medication assessed, with the exception of neuroleptic or benzodiazepine intake.

### Outcomes

Table 1 gives an overview on assessment points and outcome measures.

#### Primary outcome measure

We chose BE frequency in the last four weeks according to the *Eating* *Disorder* *Examination* *(EDE)* [48] as a primary clinical outcome. We compare changes in BE episodes at the four weeks follow-up (T8) to baseline (T0). BE frequency is of high clinical relevance as BE constitutes the core psychopathology of BED [40]. Moreover, most efficacy trials investigating psychotherapy as treatment approach look at BE frequency as primary outcome [3, 5].

#### Secondary outcome measures

Secondary outcomes will be assessed based on validated structured clinical interviews, validated self-report instruments as well as established neuropsychological tasks. The clinical BED diagnosis as well as comorbid mental disorders are assessed according to the current gold standard [42] which are standardized structured expert interviews, which are applied by trained raters. The respective assessment points can be seen in Table 1.

*Eating disorder pathology including BE frequency* as well as diagnostic criteria of BED will be assessed using the *Eating Disorder Examination* *(EDE)* [48]. Proportion of (partial) remission will be assessed according to the EDE diagnostic criteria for BED. Further, a self-developed protocol assessing BE frequency in the last seven days to assess short-term changes will be assessed [49].

*General psychopathology* will be assessed using the *Structured Clinical Interview for DSM-5 (SCID-5-CV)* to assess current and lifetime DSM-5 Axis I diagnoses of mental disorders [50]. The Beck’s Depression Inventory, Version 2 (BDI II), is used to assess depressive symptoms.

*Eating behaviour* will be assessed using the Three-factor Eating Questionnaire (TFEQ) including impulsive eating and food craving with the Food Craving Questionnaire, State Version (FCQ-S).

*Body Mass Index (BMI)* will be calculated based on objectively assessed body weight and height at T0 and T8 and assessed as self-report at T9.

*Quality of life* is assessed based on the WHO (Five)—Well-being Questionnaire (WHO-5).

*Impulsivity and Inhibitory control* will be assessed based on different approaches: On a behavior level, performance measures of the food-related antisaccade task will be analyzed, including error rate and latency [37, 38] as assessed by eye tracking (see above). Additionally, task performance in another general well-established inhibitory control task, the Go/No-Go task, will be assessed. Again, error rate and latency will be analyzed in order to estimate inhibitory control independent of disorder-relevant stimuli. Additionally, the Go/No-Go task has recently been shown to be sensitive towards changes in impulsivity in patients receiving a psychotherapy treatment [51]. Additionally, we will assess different components of self-reported impulsivity, i.e. negative urgency with the "UPPS Impulsive Behavior Scale" (UPPS), trait impulsivity with the Barratt Impulsiveness Scale (BIS-15) and common impulsive behaviours in the past seven days with a self-developed protocol [49].

*Rating of stimulus valence* of the presented food pictures is assessed based on visual analogue scales (–5 to 5) concerning valence (“very unpleasant” to “very pleasant”), appetite (“very unappetizing” to “very appetizing”), wanting (“not at all” to “very much”) and liking (“not at all” to “very much”).

*Acceptance and feasibility* will be estimated based on the percentage of included patients from the eligible patients at T0 as well as based on the drop-out rate throughout T0 to T9.

*Treatment evaluation* will be assessed using a self-developed self-report evaluation sheet which will cover aspects such as subjective need and motivation for training uptake, feasibility and acceptability of the treatment as well as overall satisfaction.

### Participant timeline

The individual participant timeline is depicted in Fig. 1.

Study duration for each patient comprises 14 weeks. This includes two weeks of training and a post-intervention follow-up period of three months. Table 1 shows the nine assessment time points during the study (T0–T9), while T0 is the baseline assessment, there is an assessment at each training session (T1–T6), a post-intervention assessment (T7) within the two weeks after training termination, a second post-intervention assessment four weeks after training termination (T8) and a three-months follow-up assessment focusing on changes in ED symptoms conducted via telephone (T9).

### Sample size

The present trial is a clinical pilot study that aims at the investigation of feasibility and effect sizes rather than significant group differences in order to establish a data basis for the preparation of a larger efficacy trial. Therefore and also due to the very limited prior evidence in the field [15, 17], a sample size calculation was not conducted.

### Randomization

Eligible patients will be randomized 1:1 between the verum stimulation and the sham stimulation. Randomization takes place after completion of the baseline assessment (T0) and is performed independently via a code system established by the Institute for Clinical Epidemiology and Applied Biostatistics, Tübingen, Germany (IKEAB). Block randomization with variable block lengths will be used.

### Blinding

ACCElect is performed as a double-blind trial. The participants as well as the data assessors are blinded regarding the treatment group. This is implemented by using a unique 5-digit code for each participant which activates either verum or sham stimulation. Moreover, also in the sham condition, tDCS electrodes are properly mounted and current is applied for 43 s at the beginning of each training session, while during this time, no inhibitory control training is conducted. Therefore, participants allocated to the sham condition perceive typical sensations of tDCS (e.g. tingling) which is considered as a valid placebo condition. To assess if blinding was successful, participants will be asked to guess in which condition they have been randomized and to indicate how certain they feel about this.

### Data management

All data is assessed pseudonymized. Data will predominantly be entered manually by either the study personnel or patients themselves for self-report measures. Data from paper-based source data will later be transferred to an electronic study database and fidelity and plausibility to the source data will be checked at random by the study personnel. All trial data will be stored in line with the European General Data Protection Regulation 2018.

### Statistical methods

The primary analysis will use the number of BE episodes in a Poisson regression model with baseline adjustment. In secondary analyses, a mixed model approach will be used to analyze the primary outcome (PO) and secondary outcomes (SOs) also with baseline adjustment. Effect sizes will be analyzed and reported for PO and SOs. From the Poisson regression, relative risks will be reported. For binary outcomes, odds ratios and for quantitative outcomes standardized differences will be reported. Primary parameters will be time vs. treatment interactions at several time points after baseline. P-values will be reported but have to be interpreted non confirmatorily. Planned, non-confirmatory subgroup analyses will be done separately for male and female subjects. The same will be done for patients receiving concomitant care vs. patients receiving no concomitant care. There will be no interim analysis. The primary analysis will be done in the intent to treat population, which is defined by including all patients with baseline assessment. Multiple imputation will be used for subjects dropping out according to the method of Rubin. The imputation model will use 500 replications and will include age, gender and baseline assessment. Furthermore, available values from preceding visits will be included. Analyses will be performed using SPSS for Windows and R.

### Ethical aspects

Ethical approval for conducting the trial has been obtained by the ethics committee of the Medical Faculty and the University Hospital Tübingen (Reference Number 459/2016BO2). All trial participants provide written informed consent prior to inclusion into the study. Patients can withdraw from the trial at any point without any disadvantage.

## Discussion

The ACCElect trial addresses the amelioration of cognitive control using a combination of an inhibitory control task with tDCS in patients suffering from BED, an ED which is characterized by impulsive eating patterns and inhibitory control deficits [7–9, 14]. The trial will provide evidence as to if NIBS approaches have the potential to improve the outcome in patients affected by BED, and specifically will inform larger RCTs focusing on efficacy in terms of effect sizes, acceptability, feasibility and safety of the combined intervention tested.

This is to the best of our knowledge one of the first studies to investigate effects of NIBS in the treatment of patients suffering from BED. Our study design has several strengths: We are investigating a clinical sample of individuals with a diagnosed BED, while it has recently been criticized that most evidence on tDCS stems from convenience and sub-clinical samples [16]. We have thoroughly pretested the study intervention and assessment methods [37, 38]. We are using an individualized training approach by adapting the food stimuli to personal preferences of each patient. We have chosen a primary outcome with high clinical relevance, which is BE frequency as core pathology of BED [40] and we have integrated a comparably long follow-up period to investigate sustainability of intervention effects while most previous evidence has exclusively looked at short-term outcomes [15, 17].

There are also several challenges for the present trial: First of all, recruitment is running during the COVID-19 pandemic, and although it was possible to implement safety measures, the pandemic impacts conduction of the trial and partly also recruitment, especially under recurrent lockdown circumstances. However, at the same time, recent data shows that people with BED are at risk of experiencing deterioration and relapse during the COVID-19 pandemic [52] and there was an overall increase in ED incidence since outbreak of COVID-19 [53], supporting the need to offer treatment to affected patients and to advance our knowledge on more effective therapy strategies.

A further challenge applying to the whole rapidly evolving research area is very limited evidence on which cognitive training approach might work best in terms of sustainable and clinically relevant changes in ED pathology. As outlined above, most previous evidence on the combination of NIBS with cognitive tasks stems from healthy convenience samples or subclinical groups [16, 18], and in the ED field, most studies have used stimulation only approaches [15, 17]. There are multiple well-validated and widely used neuropsychological paradigms to assess prefrontal brain functioning in terms of inhibitory control capacities, including the SST and GoNoGo tasks, but also the antisaccade paradigm. A currently ongoing study uses an approach bias modification training in combination with tDCS [32] as this has previously shown promise in patients with EDs. A recent meta-analysis found significant effects of tDCS on inhibitory control as assessed by the SST in healthy and in clinical samples, but not on GoNoGo performance [18]. It is also important to consider that the most widely used tasks also tap on slightly different sub-components of inhibition, for instance, the SST measures the ability to interrupt an already initiated behavior, while the GoNoGo and antisaccade paradigm assess the ability to suppress a prepotent response. These slight differences might have rather significant consequences when translating into clinical outcomes regarding targeted disorder symptoms as well as for the interplay with stimulation effects which are intended to augment the training effects [16]. Additionally, there is also the potential of delayed effects of tDCS on task performance [54], rendering it interesting for future pilot and efficacy trials to include cognitive task performance also within follow-up assessments. We aim to contribute to this evidence with our trial as more research is needed on which performance aspects are best targeted with the training as well as which aspects can be best augmented using tDCS. Therefore, we are also reporting on GoNoGo performance of our trial participants as secondary outcome.

Our main focus of the 12-week follow-up assessment are potential changes in the central clinical outcomes of the trial, while ensuring low attrition by a concise assessment. However, this was on the expense of other variables, and it would, for instance, be also insightful to have QoL data at follow-up.

An important perspective for the integration of cognitive training and NIBS into the treatment of BED is how to best combine these novel approaches with the current state-of-the-art treatments. There is recent evidence that patients with BED who received a psychotherapy for their ED [49] showed improved prefrontal cortex activity during an inhibitory control task as compared to baseline [51]. This study also showed that patients with BED with high trait impulsivity showed attenuated prefrontal cortex activation [51]. It might be speculated that a subgroup of patients characterized by severe inhibitory control deficits might specifically profit from adjunctive training + NIBS approaches prior to the first-line treatment in order to benefit most, in the sense of an enhanced psychotherapy approach. However, prior to answering these questions on optimal sequence and tailoring of treatment modules, larger efficacy trials on the usefulness of the present combination treatment are needed.

We hope that data from the ACCElect trial will contribute to the development of novel neurobiologically informed treatment approaches for patients suffering from BED and to support this patient group in achieving more sustainable abstinence from binge-eating.

## Data Availability

Not applicable.

## References

[1] Hoek HW (2016). Review of the worldwide epidemiology of eating disorders. Curr Opin Psychiatry.

[2] Keski-Rahkonen A, Mustelin L (2016). Epidemiology of eating disorders in Europe: prevalence, incidence, comorbidity, course, consequences, and risk factors. Curr Opin Psychiatry.

[3] Hilbert A, Petroff D, Herpertz S, Pietrowsky R, Tuschen-Caffier B, Vocks S (2019). Meta-analysis of the efficacy of psychological and medical treatments for binge-eating disorder. J Consult Clin Psychol.

[4] National Institute for Health and Care Excellence (NICE). Eating Disorders: recognition and treatment Full guideline 2017. Available from: https://www.nice.org.uk/guidance/ng69/evidence/full-guideline-pdf-161214767896. 28654225

[5] Hilbert A, Petroff D, Herpertz S, Pietrowsky R, Tuschen-Caffier B, Vocks S (2020). Meta-analysis on the long-term effectiveness of psychological and medical treatments for binge-eating disorder. Int J Eat Disord.

[6] Santomauro DF, Melen S, Mitchison D, Vos T, Whiteford H, Ferrari AJ (2021). The hidden burden of eating disorders: an extension of estimates from the Global Burden of Disease Study 2019. Lancet Psychiatry.

[7] Kessler RM, Hutson PH, Herman BK, Potenza MN (2016). The neurobiological basis of binge-eating disorder. Neurosci Biobehav Rev.

[8] Schag K, Schonleber J, Teufel M, Zipfel S, Giel KE (2013). Food-related impulsivity in obesity and binge eating disorder–a systematic review. Obes Rev.

[9] Giel KE, Teufel M, Junne F, Zipfel S, Schag K. Food-related impulsivity in obesity and binge eating disorder: a systematic update of the evidence. Nutrients. 2017;9(11). 10.3390/nu9111170 PMC5707642 29077027

[10] Miranda-Olivos R, Steward T, Martinez-Zalacain I, Mestre-Bach G, Juaneda-Segui A, Jimenez-Murcia S, et al. The neural correlates of delay discounting in obesity and binge eating disorder. J Behav Addict. 2021. 10.1556/2006.2021.00023 PMC8997223 33950859

[11] Iceta S, Rodrigue C, Legendre M, Daoust J, Flaudias V, Michaud A, et al. Cognitive function in binge eating disorder and food addiction: a systematic review and three-level meta-analysis. Prog Neuropsychopharmacol Biol Psychiatry. 2021;111:110400. 10.1016/j.pnpbp.2021.110400 34256024

[12] Leehr EJ, Krohmer K, Schag K, Dresler T, Zipfel S, Giel KE (2015). Emotion regulation model in binge eating disorder and obesity: a systematic review. Neurosci Biobehav Rev.

[13] Cole MW, Schneider W (2007). The cognitive control network: Integrated cortical regions with dissociable functions. Neuroimage.

[14] Lavagnino L, Arnone D, Cao B, Soares JC, Selvaraj S (2016). Inhibitory control in obesity and binge eating disorder: a systematic review and meta-analysis of neurocognitive and neuroimaging studies. Neurosci Biobehav Rev.

[15] Dalton B, Campbell IC, Schmidt U (2017). Neuromodulation and neurofeedback treatments in eating disorders and obesity. Curr Opin Psychiatry.

[16] Chase HW, Boudewyn MA, Carter CS, Phillips ML (2020). Transcranial direct current stimulation: a roadmap for research, from mechanism of action to clinical implementation. Mol Psychiatry.

[17] Dalton B, Bartholdy S, Campbell IC, Schmidt U (2018). Neurostimulation in clinical and sub-clinical eating disorders: a systematic update of the literature. Curr Neuropharmacol.

[18] Schroeder PA, Schwippel T, Wolz I, Svaldi J (2020). Meta-analysis of the effects of transcranial direct current stimulation on inhibitory control. Brain Stimul.

[19] İnce B, Schlatter J, Max S, Plewnia C, Zipfel S, Giel KE (2021). Can we change binge eating behaviour by interventions addressing food-related impulsivity? A systematic review. J Eat Disord.

[20] Burgess EE, Sylvester MD, Morse KE, Amthor FR, Mrug S, Lokken KL (2016). Effects of transcranial direct current stimulation (tDCS) on binge eating disorder. Int J Eat Disord.

[21] Fritsch B, Reis J, Martinowich K, Schambra HM, Ji Y, Cohen LG (2010). Direct current stimulation promotes BDNF-dependent synaptic plasticity: potential implications for motor learning. Neuron.

[22] Kronberg G, Bridi M, Abel T, Bikson M, Parra LC (2017). Direct current stimulation modulates LTP and LTD: activity dependence and dendritic effects. Brain Stimul.

[23] Gill J, Shah-Basak PP, Hamilton R (2015). It's the thought that counts: examining the task-dependent effects of transcranial direct current stimulation on executive function. Brain Stimul.

[24] Zwissler B, Sperber C, Aigeldinger S, Schindler S, Kissler J, Plewnia C (2014). Shaping memory accuracy by left prefrontal transcranial direct current stimulation. J Neurosci.

[25] Boroda E, Sponheim SR, Fiecas M, Lim KO. Transcranial direct current stimulation (tDCS) elicits stimulus-specific enhancement of cortical plasticity. Neuroimage. 2020;211:116598. 10.1016/j.neuroimage.2020.116598 32032738

[26] Bikson M, Name A, Rahman A (2013). Origins of specificity during tDCS: anatomical, activity-selective, and input-bias mechanisms. Front Hum Neurosci.

[27] Sathappan AV, Luber BM, Lisanby SH (2019). The dynamic duo: combining noninvasive brain stimulation with cognitive interventions. Prog Neuropsychopharmacol Biol Psychiatry.

[28] Segrave RA, Arnold S, Hoy K, Fitzgerald PB (2014). Concurrent cognitive control training augments the antidepressant efficacy of tDCS: a pilot study. Brain Stimul.

[29] Brunoni AR, Boggio PS, De Raedt R, Bensenor IM, Lotufo PA, Namur V (2014). Cognitive control therapy and transcranial direct current stimulation for depression: a randomized, double-blinded, controlled trial. J Affect Disord.

[30] Plewnia C, Schroeder PA, Wolkenstein L (2015). Targeting the biased brain: non-invasive brain stimulation to ameliorate cognitive control. Lancet Psychiatry.

[31] Schwippel T, Papazova I, Strube W, Fallgatter AJ, Hasan A, Plewnia C (2018). Beneficial effects of anodal transcranial direct current stimulation (tDCS) on spatial working memory in patients with schizophrenia. Eur Neuropsychopharmacol.

[32] Gordon G, Brockmeyer T, Schmidt U, Campbell IC. Combining cognitive bias modification training (CBM) and transcranial direct current stimulation (tDCS) to treat binge eating disorder: study protocol of a randomised controlled feasibility trial. BMJ Open. 2019;9(10):e030023. 10.1136/bmjopen-2019-030023 PMC6830595 31640997

[33] Brevet-Aeby C, Brunelin J, Iceta S, Padovan C, Poulet E (2016). Prefrontal cortex and impulsivity: interest of noninvasive brain stimulation. Neurosci Biobehav Rev.

[34] Sauvaget A, Trojak B, Bulteau S, Jimenez-Murcia S, Fernandez-Aranda F, Wolz I (2015). Transcranial direct current stimulation (tDCS) in behavioral and food addiction: a systematic review of efficacy, technical, and methodological issues. Front Neurosci.

[35] Jansen JM, Daams JG, Koeter MW, Veltman DJ, van den Brink W, Goudriaan AE (2013). Effects of non-invasive neurostimulation on craving: a meta-analysis. Neurosci Biobehav Rev.

[36] Maranhao MF, Estella NM, Cury ME, Amigo VL, Picasso CM, Berberian A (2015). The effects of repetitive transcranial magnetic stimulation in obese females with binge eating disorder: a protocol for a double-blinded, randomized, sham-controlled trial. BMC Psychiatry.

[37] Max SM, Plewnia C, Zipfel S, Giel KE, Schag K (2021). Combined antisaccade task and transcranial direct current stimulation to increase response inhibition in binge eating disorder. Eur Arch Psychiatry Clin Neurosci.

[38] Giel KE, Speer E, Schag K, Leehr EJ, Zipfel S (2017). Effects of a food-specific inhibition training in individuals with binge eating disorder-findings from a randomized controlled proof-of-concept study. Eat Weight Disord.

[39] Schag K, Teufel M, Junne F, Preissl H, Hautzinger M, Zipfel S, et al. Impulsivity in binge eating disorder: food cues elicit increased reward responses and disinhibition. PLoS One. 2013;8(10):e76542. 10.1371/journal.pone.0076542 PMC3797795 24146885

[40] American Psychiatric Association, editor. Diagnostic and statistical manual of mental disorders: DSM-5. Washington, DC: American Psychiatric Assoc.; 2013.

[41] Chan AW, Tetzlaff JM, Altman DG, Laupacis A, Gotzsche PC, Krleza-Jeric K (2013). SPIRIT 2013 statement: defining standard protocol items for clinical trials. Ann Intern Med.

[42] American Psychological Association (APA). Practice Guideline for the Treatment of Patients with Eating Disorders 2006. Available from: https://psychiatryonline.org/pb/assets/raw/sitewide/practice_guidelines/guidelines/eatingdisorders.pdf.

[43] Hutton SB, Ettinger U (2006). The antisaccade task as a research tool in psychopathology: a critical review. Psychophysiology.

[44] Leehr EJ, Schag K, Dresler T, Grosse-Wentrup M, Hautzinger M, Fallgatter AJ (2018). Food specific inhibitory control under negative mood in binge-eating disorder: evidence from a multimethod approach. Int J Eat Disord.

[45] Jasper H (1958). The ten-twenty electrode system of the International Federation. Electroencephalogr Clin Neurophysiol.

[46] Nasseri P, Nitsche MA, Ekhtiari H (2015). A framework for categorizing electrode montages in transcranial direct current stimulation. Front Hum Neurosci.

[47] Paulus W (2003). Transcranial direct current stimulation (tDCS). Suppl Clin Neurophysiol.

[48] Hilbert A, Tuschen-Caffier B. Eating Disorder Examniation. Tübingen: dgvt-Verlag; 2016.

[49] Schag K, Rennhak SK, Leehr EJ, Skoda EM, Becker S, Bethge W (2019). IMPULS: impulsivity-focused group intervention to reduce binge eating episodes in patients with binge eating disorder—a randomised controlled trial. Psychother Psychosom.

[50] Beesdo-Baum K, Zaudig M, Wittchen H. Strukturiertes Klinisches Interview für DSM-5®-Störungen – Klinische Version Göttingen: Hogrefe; 2019.

[51] Veit R, Schag K, Schopf E, Borutta M, Kreutzer J, Ehlis AC, et al. Diminished prefrontal cortex activation in patients with binge eating disorder associates with trait impulsivity and improves after impulsivity-focused treatment based on a randomized controlled IMPULS trial. Neuroimage Clin. 2021;30:102679. 10.1016/j.nicl.2021.102679 PMC8102655 34215149

[52] Giel KE, Schurr M, Zipfel S, Junne F, Schag K (2021). Eating behaviour and symptom trajectories in patients with a history of binge eating disorder during COVID-19 pandemic. Eur Eat Disord Rev.

[53] Taquet M, Geddes J, Luciano S, Harrison P. Incidence and outcomes of eating disorders during the COVID-19 pandemic. Br J Psychiatry. 2021. 10.1192/bjp.2021.105 PMC7612698 35048812

[54] Martin DM, Mohan A, Alonzo A, Gates N, Gbadeyan O, Meinzer M (2019). A pilot double-blind randomized controlled trial of cognitive training combined with transcranial direct current stimulation for amnestic mild cognitive impairment. J Alzheimers Dis.

